# 

**Published:** 1844-06

**Authors:** 


### CONTENTS

OF NO. VI.

### ORIGINAL COMMUNICATIONS.

ESSAYS AND CASES.

ART. I.—Experimental Researches upon Febrile Caloricity, both before and after death—Post-mortem Fever. By BENNET DOWLER, M.D., of New Orleans. - - - - - - - - - 469
ART. II.—A Case of angular Anchylosis of the Knee Joint, treated on Dr. J. R. Barton's plan by PLATT BURR, M.D. Reported by ANDREW R. KILPATRICK, M.D., of Woodville, Mississippi. - 502

REVIEWS.

ART. III.—A Treatise on the nature, cause, and treatment of Erysipelas. By THOS. NUNNELEY, Lecturer on Anatomy, Physiology, and Pathology, in the Leeds Medical School; &c. &c. - - 507
ART. IV.—A Practical Treatise on Midwifery. By M. CHAILLY, M.D., and Ex-Chief of the Obstetric Clinique of the Faculty of Paris; Professor of Midwifery, &c., &c., &c. Illustrated with 216 wood-cuts. Translated from the French and Edited by GUNNING S. BEDFORD, M.D., &c. - - 513
ART. V.—The Cyclopœdia of Practical Medicine. Edited by JOHN FORBES, M.D., F.R.S., ALEX. TWEEDIE, M.D., F.R.S., and JOHN CONNOLLY, M.D. Revised, with additions, by ROBLEY DUNGLISON, M.D. 514

Art. VI.—Transactions of the N. York State Medical Society. Vol. vi. Part i. - - - - - 514

SELECTIONS FROM AMERICAN AND FOREIGN JOURNALS.

Employment of Electricity and Electro-Magnetism in Poisoning by Laudanum. - - - - - - 517
Open Anus in certain forms of Fæcal Incontinence. - - 521
Progress of Thomsonism. - - - - - - - 522
Disgraceful Advertisements. - - - - - - 522
Intemperate Physicians. - - - - - - - 523
Naphtha in Pulmonary Consumption. - - - 523
Remarkable case of Abdominal Tumor. - - - 527
Tic Douloureux of the Face and Head. - - - 532
Employment of Chloride of Zinc in Toothache. - - 532
Animalcules in the Intestinal Canal. - - - - 533
Chlorosis. - - - - - - - - - - 534
Sir B. C. Brodie on Fistula in Ano. - - - 535

ORIGINAL INTELLIGENCE.

Traveling Letters. - - - - - - - - 546
Illinois Medical Journal. - - - - - - - 554
Professor Bartlett. - - - - - - - - 555
Medical Society of Tennessee. - - - - - 555
Professor Caldwell and Animal Chemistry. - - 556
Professor Hall, of Baltimore. - - - - - - 556
Necrology. - - - - - - - - - 556

